# A cross-sectional study of contact allergens in feminine hygiene wipes: a possible cause of vulvar contact dermatitis

**DOI:** 10.1097/JW9.0000000000000060

**Published:** 2022-11-28

**Authors:** Jazmin Newton, Sophie Richardson, Annika M. van Oosbre, Jiade Yu, Channi Silence

**Affiliations:** a University of South Dakota Sanford School of Medicine, Vermillion, South Dakota; b Department of Dermatology, Harvard Medical School/Massachusetts General Hospital, Boston, Massachusetts; c Tufts University School of Medicine, Boston, Massachusetts

## Abstract

Feminine hygiene wipes marketed toward women for maintaining freshness and cleanliness of the vulva and perineum are abundant both in-store and online. Many of these products boast being “fragrance free,” “gentle,” and “for sensitive skin,” which is attractive to consumers. However, these claims do not necessarily mean they are free of potential allergens.

**Objective:**

The present study aims to investigate the presence and prevalence of potential allergens in the most used feminine hygiene wipes.

**Methods:**

An internet-based search was performed to identify best-selling name brand and generic feminine hygiene wipes. Each unique wipe was analyzed and compared to the North American Contact Dermatitis Group 80 allergens.

**Results:**

We found contact allergens are frequently present in feminine hygiene wipes, most commonly fragrances, other scented botanicals in the form of essences, oils, and fruit juices, and vitamin E (tocopherol). All wipes analyzed in this study contained potential allergens.

**Limitations:**

The inability to eliminate commercial names from analysis could have introduced bias.

**Conclusions:**

Vaginal and vulvar epithelia are highly susceptible to contact allergens, often found in products marketed for feminine hygiene and cleanliness. Providers should caution patients against trusting product labeling claims to avoid incidental contact allergy and encourage simply cleansing the vulva with water.

**What is known about this subject in regard to women and their families?**

- Feminine hygiene wipes sold in the United States contain numerous allergens.
- Products labeled as “natural,” “sensitive,” or “gentle” still contain allergens.
- Vaginal and vulvar epithelia are highly susceptible to contact allergens.
- Clinical features of vulvar contact dermatitis include erythema, swelling, scaling, excoriations, fissures, erosions, and ulcers.

**What is new from this article as messages for women and their families?**

- Patients should be cautioned against trusting labeling claims that a product is “natural,” “fragrance-free,” “soothing,” “organic,” and the like, as products with such labels still contain potential contact allergens.
- Encourage patients to clean their vulvas using only water per the American College of Obstetrics and Gynecology recommendations.

## Introduction

Allergic contact dermatitis (ACD) is a type IV cell-mediated delayed-type hypersensitivity reaction that affects 20% of general adult and pediatric populations.^1,2^ Personal care products frequently contain allergens that may elicit ACD.

The vulvar and vaginal skin is unique due to its nonkeratinized nature and elevated hydration and frictional properties. Therefore this area is much more susceptible to these allergens than keratinized skin.^3^ Vulvar contact dermatitis, which is caused by allergens irritating the vulva, poses a significant issue for patients. Vulvar pruritus is a common chief complaint, with many patients experiencing sexual ramifications. Patients may feel insecure participating in intercourse and struggle to enjoy it, given the pruritic sensation they are also experiencing. Additionally, patients may find the sensation distressing and distracting in their everyday lives.

Wet wipes that are marketed as maintaining freshness and cleanliness of the vulva and perineum are popular. Many of these products boast being “fragrance free,” “gentle,” “for sensitive skin,” and “non-irritating,” which is often attractive to consumers. However, these claims are seldom based on evidence, and a review of popular feminine hygiene wipes (FHW) ingredients has not been performed. The aim of this study is to investigate the presence and prevalence of potential allergens in popular FHW.

## Methods

**Fig. 1.**
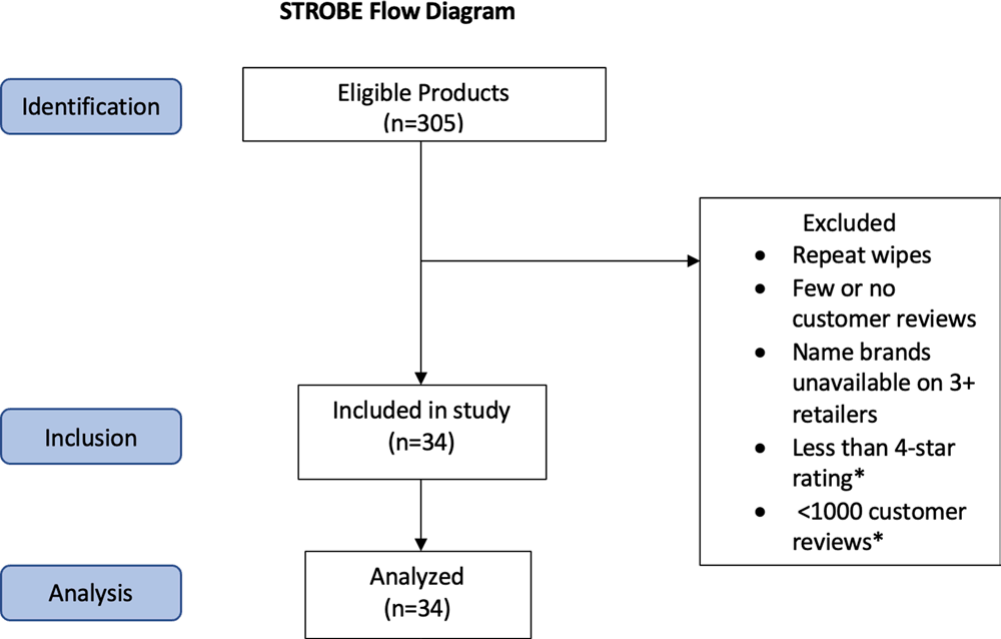
Product selection flowchart. STROBE, strengthening the reporting of observational studies in epidemiology.

An internet-based search of 5 major retailers including Walmart, Target, CVS, Walgreens, and Amazon was performed to identify current best-selling name brand and generic FHWs marketed to women in the United States for the purpose of maintaining hygienic cleanliness or freshness of the genital area. To identify products, each retailer website was searched using both of the following terms: “feminine wipes” and “feminine hygiene wipes.” Those wipes available on each retailer website were included, excluding wipes that were of the same brand that varied only by scent, those that had few or no customer reviews compared to other wipes on that site, and/or those name brands that were not available on at least 2 other retailer websites. Additionally, given the expansive nature of Amazon, products without at least a 4-star rating and >1000 customer reviews were excluded. Twenty-eight different brands yielding 34 unique hygienic wipe products advertised for sweat, sex, menstrual cycle, and/or home/public bathroom use were selected for review (Fig. 1). The manufacturer ingredient list for each unique hygienic wipe was analyzed and compared to the North American Contact Dermatitis Group (NACDG) 80 allergens.^4^

The mean number of allergens was calculated for all products combined. This was further divided into the mean number of allergens in FHWs marketed as “sensitive,” “natural,” “fragrance-free,” and so forth, and those not marketed this way. The difference in number of allergens between these groups was calculated using a 2-tailed t-test.

## Results

**Fig. 2.**
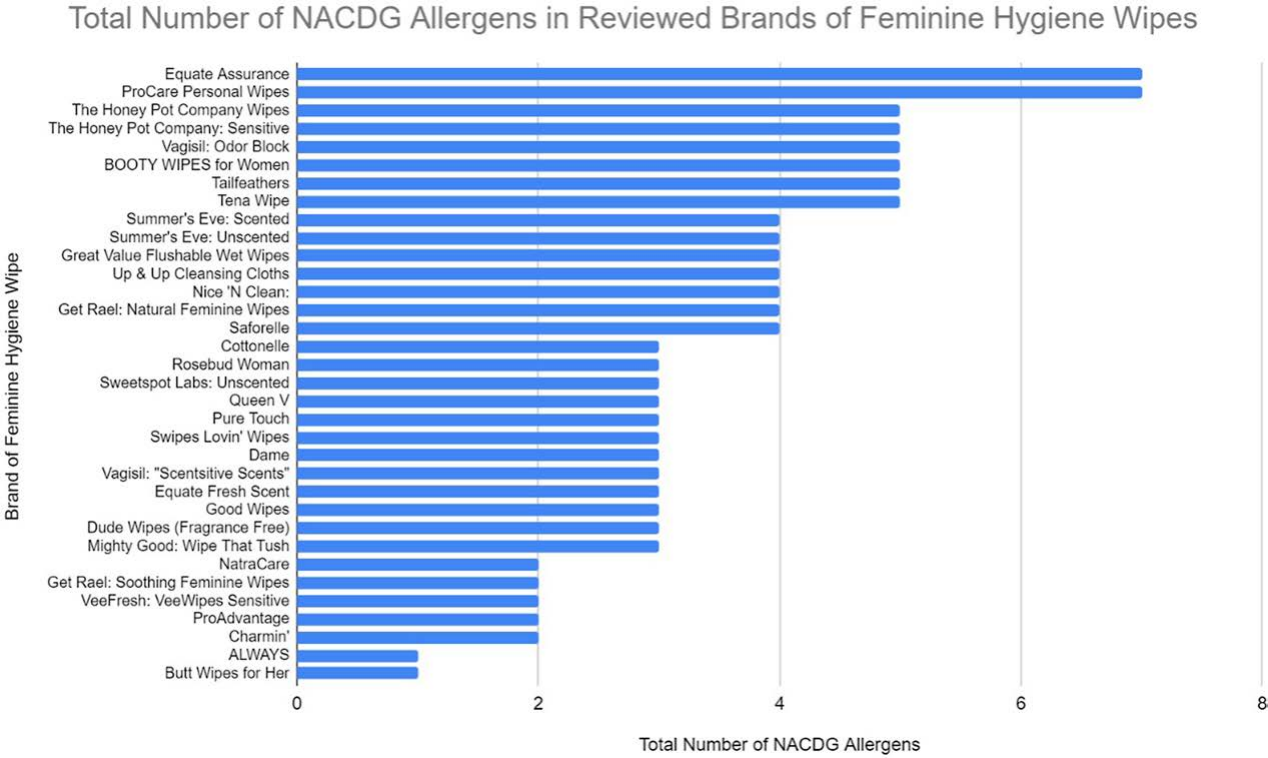
Total number of screened NACDG allergens per feminine hygiene wipe. NACDG, North American Contact Dermatitis Group.

The most common sensitizers in the products analyzed were fragrances and tocopherol, both of which were found in 17 of the 34 products (50%). Botanicals including extracts, oils, and fruit juices were used in 10 products (Fig. 2).

**Fig. 3.**
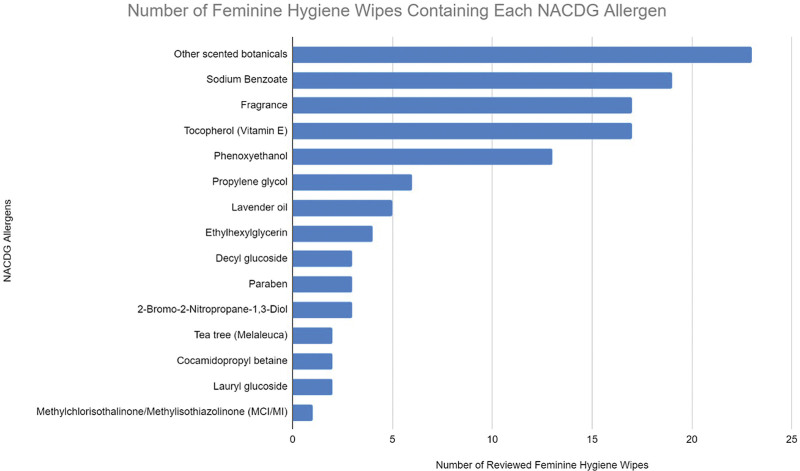
Number of feminine hygiene wipes containing each NACDG allergen. Allergens listed in none of the ingredient lists are not included. NACDG, North American Contact Dermatitis Group.

Every product analyzed contained at least one possible allergen, with a mean of 3.53 allergens per product (Table 1). Products with the highest numbers of allergens include Equate Assurance and ProCare Personal Wipes. These both contained 6 allergens. Twenty-seven of the 34 products analyzed (76.5%) contained 3 or more allergens. Two products tied for the lowest number of allergens with one present, ALWAYS Feminine Wipes and Butt Wipes for Her, which contained fragrance and tocopherol, respectively (Fig. 3).

**Table 1:** Prevalence of screened NACDG allergens in feminine hygiene wipes

No. allergens	0	≥1	≥2	≥3	≥4	≥5	≥6
No. products	0	34	32	27	15	8	2

NACDG, North American Contact Dermatitis Group.

Of note, 9 products were advertised as “sensitive,” “natural,” “fragrance free,” “soothing,” or “organic.” Products with these claims did not have a statistically significant difference in total number of allergens compared to products that did not make these claims (mean of 3.11 vs 3.68, *P* = 0.31). The Summer’s Eve brand scented versus unscented products contained the same number of allergens (4)—fragrance was exchanged for botanicals in the unscented version. Honey Pot sensitive versus regular also both had the same number of allergens (5); the sensitive product had fragrance, whereas the regular listed lavender oil instead.

## Discussion

The present study illustrates that all FHWs contain potential allergens such as those commonly tested for by the NACDG.

Fragrances were the most common allergen in the FHWs analyzed in the present study, with 50% of products containing it. Fragrances, essential oils, and botanicals have also been found to be the most common allergen in various personal care products, including laundry detergents (66.7%), fabric softeners (90%), dryer sheets (75%), stain removers (58.8%), facial wipes (63.5%), and personal care products for babies/children (47.8%).^5–7^ Fragrance mix I, Balsam of Peru, and fragrance mix II are in the top 15 most common contact allergens in the most recent NACDG data accounting for 9.2%, 7.1%, and 4.4% of positive patch test reactions, respectively.^4,8–13^ In fact, fragrance was named allergen of the year by the ACDS in 2007, with ten percent of patients with atopic dermatitis testing positive for fragrance upon patch testing.^14^

The other most common allergen in the present study was tocopherol, which was also found in 50% of the studied products. This compound acts as a moisturizer, antioxidant, and antiaging agent by absorbing ultraviolet wavelengths to prevent oxidative stress; yet, it is also considered an allergen.^15–17^ However, positive patch test reactions only occurred in 0.6% of 4859 patients in the NACDG patch test results.^4^ Many case reports have been published citing tocopherol-induced ACD, though many are without confirmation by patch testing.^15^ A review of the published literature on tocopherol-induced ACD found that it is overall uncommon despite its frequent use in skin care products. Additionally, this review noted that higher concentrations of tocopherol trigger ACD more often than lower concentrations^15,18^

Products advertised as “fragrance free” still may include fragrance products as preservatives or botanical add-ins.^14^ The present study did not identify a significant difference in the total number of possible sensitizing agents between products labeled as “natural,” “fragrance free,” “soothing,” and so forth, and those that were not across different brands. This is not an unusual trend—methylisothiazolinone was identified in 57.1% of “free and gentle” laundry detergents and 19.7% of pediatric skin care products labeled “gentle,” “sensitive,” or “hypoallergenic.”^6,19^ In fact, there are no regulations/mandates in place to govern these claims.^19^ Of note, in the present study, products with these labels did occasionally contain fewer allergens (eg, Vagisil “Sensitive Scents” contained 3 allergens vs 5 in Vagisil Odor Block). However, other brands such as Summer’s Eve and The Honey Pot merely exchanged fragrance for extracts/oils in their unscented or sensitive products.

The American College of Obstetricians and Gynecologists (ACOG) recommends a systematic approach to patients presenting with vulvovaginal symptoms. Differentiating between vulvar pruritus and pain is a key first distinction; pruritus should be then categorized as either acute or chronic. The presence of abnormal discharge should be investigated for an infectious etiology, including bacterial vaginosis, trichomoniasis, and candidiasis. Other potential infectious etiologies causing pruritus include tinea cruris, molluscum contagiosum, and scabies/pediculosis. After ruling these out, dermatoses, including ACD, should be considered. A thorough history of potential contact allergens should be delineated. Clinical features include erythema, swelling, scaling, excoriations, fissures, erosions, and ulcers.^20,21^ Patients should be counseled to avoid known vulvar irritants. In addition to avoidance, treatment for ACD consists of a topical corticosteroid product applied once to twice daily and possibly an oral antipruritic medication such as an antihistamine.^20,22^ Topical antipruritic medications can cause allergic dermatitis, so they should not be used.^23^ Patients should be counseled to cleanse the vulva with water only in the future, even after intercourse.^24^

Patients should be cautioned against trusting labeling claims that a product is “natural,” “fragrance-free,” “soothing,” “organic,” and the like. Specifically, we recommend advising patients to look for fragrance, oils, and other botanicals on product labels, as these are easy-to-identify potential allergens. Additionally, we recommend all providers encourage patients to clean their vulvas using simply water, per ACOG practice bulletin recommendations.^24^

The main limitation of our study is that we could not eliminate commercial names from our analysis, introducing potential bias. Future research could investigate contact allergens in other feminine hygiene products, including body wash, menstrual pads, and tampons, in addition to sex products including lubricants.

## Conclusions

Vaginal and vulvar epithelia are highly susceptible to contact allergens, many of which are found in products marketed towards feminine hygiene and cleanliness of the vulva and perineum. This study reviewed 34 of the top-selling feminine hygiene/intimate wipes for 80 contact allergens identified by the NACDG. Contact allergens were found in every single product reviewed, the most common being fragrances, other scented botanicals, and tocopherol. The popularity of these products indicates a need for improved allergen identification, product labeling, and consumer education regarding vulvar hygiene.

## Conflicts of Interest

None.

## Funding

None.

## Study approval

N/A.

## References

[1] Jacob SE Steele T Brod B. Dispelling the myths behind pediatric patch testing-experience from our tertiary care patch testing centers. Pediatr Dermatol 2008;25:296–300. 18577031 10.1111/j.1525-1470.2008.00670.x

[2] Thyssen JP Linneberg A Menne T. The epidemiology of contact allergy in the general population--prevalence and main findings. Contact Dermatitis 2007;57:287–299. 17937743 10.1111/j.1600-0536.2007.01220.x

[3] Farage MA. Vulvar susceptibility to contact irritants and allergens: a review. Arch Gynecol Obstet 2005;272:167–172. 15906051 10.1007/s00404-005-0732-4

[4] DeKoven JG Silverberg JI Warshaw EM. North American Contact Dermatitis Group patch test results: 2017-2018. Dermatitis 2021;32:111–123. 33970567 10.1097/DER.0000000000000729

[5] Aschenbeck KA Warshaw EM. Allergenic ingredients in facial wet wipes. Dermatitis 2017;28:353–359. 28338538 10.1097/DER.0000000000000268

[6] Bai H Tam I Yu J. Contact allergens in top-selling textile-care products. Dermatitis 2020;31:53–58. 31905182 10.1097/DER.0000000000000566

[7] Bonchak JG Prouty ME de la Feld SF. Prevalence of contact allergens in personal care products for babies and children. Dermatitis 2018;29:81–84. 29494392 10.1097/DER.0000000000000348

[8] Fransway AF Zug KA Belsito DV. North American Contact Dermatitis Group patch test results for 2007-2008. Dermatitis 2013;24:10–21. 23340394 10.1097/DER.0b013e318277ca50

[9] Warshaw EM Belsito DV Taylor JS. North American Contact Dermatitis Group patch test results: 2009 to 2010. Dermatitis 2013;24:50–59. 23474444 10.1097/DER.0b013e3182819c51

[10] Warshaw EM Maibach HI Taylor JS. North American Contact Dermatitis Group patch test results: 2011-2012. Dermatitis 2015;26:49–59. 25581671 10.1097/DER.0000000000000097

[11] Wetter DA Davis MD Yiannias JA. Patch test results from the mayo clinic contact dermatitis group, 1998-2000. J Am Acad Dermatol 2005;53:416–421. 16112346 10.1016/j.jaad.2005.04.077

[12] Wetter DA Yiannias JA Prakash AV. Results of patch testing to personal care product allergens in a standard series and a supplemental cosmetic series: an analysis of 945 patients from the Mayo Clinic Contact Dermatitis Group, 2000-2007. J Am Acad Dermatol 2010;63:789–798. 20643495 10.1016/j.jaad.2009.11.033

[13] Zug KA Warshaw EM Fowler JF Jr.. Patch-test results of the North American Contact Dermatitis Group 2005-2006. Dermatitis 2009;20:149–160. 19470301

[14] Johansen JD. Fragrance contact allergy: a clinical review. Am J Clin Dermatol 2003;4:789–798. 14572300 10.2165/00128071-200304110-00006

[15] Kosari P Alikhan A Sockolov M. Vitamin E and allergic contact dermatitis. Dermatitis 2010;21:148–153. 20487657

[16] Krol ES Kramer-Stickland KA Liebler DC. Photoprotective actions of topically applied vitamin E. Drug Metab Rev 2000;32:413–420. 11139138 10.1081/dmr-100102343

[17] Manela-Azulay M Bagatin E. Cosmeceuticals vitamins. Clin Dermatol 2009;27:469–474. 19695478 10.1016/j.clindermatol.2009.05.010

[18] Roed-Petersen J Hjorth N. Patch test sensitization from d,l-alpha-tocopherol (vitamin E). Contact Dermatitis 1975;1:391. 1235304

[19] Schlichte MJ Katta R. Methylisothiazolinone: an emergent allergen in common pediatric skin care products. Dermatol Res Pract 2014;2014:132564. 25342949 10.1155/2014/132564 PMC4197884

[20] Margesson LJ. Contact dermatitis of the vulva. Dermatol Ther 2004;17:20–27. 14756887 10.1111/j.1396-0296.2004.04003.x

[21] Stockdale CK Boardman L. Diagnosis and treatment of vulvar dermatoses. Obstet Gynecol 2018;131:371–386. 29324620 10.1097/AOG.0000000000002460

[22] van der Meijden WI Boffa MJ Ter Harmsel WA. 2016 European guideline for the management of vulval conditions. J Eur Acad Dermatol Venereol 2017;31:925–941. 28164373 10.1111/jdv.14096

[23] Kellogg Spadt S Kusturiss E. Vulvar dermatoses: a primer for the sexual medicine clinician. Sex Med Rev 2015;3:126–136. 27784605 10.1002/smrj.55

[24] ACOG. Diagnosis and Management of Vulvar Skin Disorders. Obstetr Gynecol 2020;136:e1–e14. 10.1097/AOG.0000000000003944 32590724

